# Draft Genome Sequence of *Methylobacterium* sp. Strain L2-4, a Leaf-Associated Endophytic N-Fixing Bacterium Isolated from *Jatropha curcas* L.

**DOI:** 10.1128/genomeA.01306-14

**Published:** 2014-12-11

**Authors:** Munusamy Madhaiyan, Kam Lock Chan, Lianghui Ji

**Affiliations:** Biomaterials and Biocatalysts Group, Temasek Life Sciences Laboratory, National University of Singapore, Singapore

## Abstract

*Methylobacterium* sp. strain L2-4 is an efficient nitrogen-fixing leaf colonizer of biofuel crop *Jatropha curcas*. This strain is able to greatly improve the growth and seed yield of *Jatropha curcas* and is the second reported genome sequence of plant growth-promoting bacteria isolated from *Jatropha curcas*.

## GENOME ANNOUNCEMENT

As biofuel crop *Jatropha curcas* is targeted to marginal land where soil nutrient is low, the requirement for nitrogen fertilizer will be higher than other crops. The use of plant growth-promoting bacteria is a promising approach to improve productivity and the green index of *Jatropha* biodiesel (1–3). *Methylobacterium* species are abundant on plant leaf tissues as endophytes or epiphytes on leaves (4–11) and exert a positive effect on the growth and development of plants by playing a role in seed germination and root development, drought tolerance, growth promotion, and increasing the yield of agricultural plants (12, 13). Previously, some *Methylobacterium* strains have been found in symbiotic association with *Crotalaria* and *Lotononis*, both legumes where the *Methylobacterium* induces nodulation and fix nitrogen in the nodules (14, 15). We have identified an efficient nitrogen-fixing leaf-colonizer, strain L2-4 belonging to *Methylobacterium*, from surface-sterilized leaf tissues of *Jatropha curcas*. It is capable of fixing nitrogen to about 634.5 nmol C_2_H_4_ released h^−1^ bottle^−1^ as measured by Hardy’s acetylene method (16, 17).

Genome sequencing was carried out using the GS FLX Titanium platform at Macrogen, Inc. (Republic of Korea). The sequence reads were assembled using the GS De Novo Assembler (v2.6). The genome was annotated using the Rapid Annotations using Subsystems Technology server employing the GLIMMER gene caller (18). The draft genome sequence was also annotated using the NCBI Prokaryotic Genome Annotation Pipeline (PGAAP) (http://www.ncbi.nlm.nih.gov/genome/annotation_prok/). The draft genome sequence of strain L2-4 included 342 contigs (>500 bp in size), with a calculated genome size 6,800,472 bp long, containing 6,382 protein-coding genes and an average G+C content of 70.8%. A total of 6,255 genes were assigned though the PGAAP and categorized into 6,092 coding sequences, 103 pseudogenes, 5 rRNAs (16S, 23S), 54 tRNAs, 1 noncoding RNA (ncRNA), and 87 frameshifted genes. Comparison of its 16S rRNA genes with EzGenome (http://ezgenome.ezbiocloud.net/) using BLASTn revealed that it shares the highest nucleic acid identity with the UV-resistant *Methylobacterium radiotolerans* JCM 2831 (99%), followed by *Methylobacterium* sp. GXF4 (98%) and *Methylobacterium extorquens* AM1 (95%).

Strain L2-4 possesses a conserved cluster of genes associated with photosynthesis, including genes encoding the light-harvesting complex and the reaction center, and genes involved in biosynthesis of bacteriochlorophyll (*bch*) and carotenoids (*crt*). Further analyses of this genome will include comparisons with other *Methylobacterium* genomes already reported (13, 17, 19–21). The genome of strain L2-4 presents several genes involved in metabolic pathways that may contribute to the promotion of plant growth, including genes for the production of auxin biosynthesis, zeatin (*miaA*), cobalamin synthesis protein (*cob*), urea metabolism (*ureABCDEFG*), biosorption of heavy metals or decrease of metal toxicity, endoglucanase (*celC*), phytase, C-P lyase system (*phn*), pyrroloquinoline quinone biosynthesis protein (*pqqABCDE*), and methylotrophy gene clusters (*mxa*). In addition, the gene coding for the 1-aminocyclopropane-1-carboxylate deaminase (*acdS*) gene is also observed, which may suggest contributions to plant development under stress conditions (22). The genome information presented here will allow in-depth functional and comparative genome analyses to provide a better understanding of beneficial plant-bacterial associations.

### Nucleotide sequence accession numbers.

This whole-genome shotgun project has been deposited at DDBJ/EMBL/GenBank under the accession no. AVNX00000000. The version described in this paper is version AVNX01000000.
